# The pan-JAK inhibitor LAS194046 reduces neutrophil activation from severe asthma and COPD patients in vitro

**DOI:** 10.1038/s41598-022-09241-6

**Published:** 2022-03-24

**Authors:** Javier Milara, Beatriz Ballester, Alfredo de Diego, Marta Calbet, Isabel Ramis, Montserrat Miralpeix, Julio Cortijo

**Affiliations:** 1 Department of Pharmacology, Faculty of Medicine, University of Valencia, Valencia, Spain; 2 Pharmacy Unit, Consorcio Hospital General Universitario, Avenida tres cruces s/n, 46014 Valencia, Spain; 3 CIBERES, Health Institute Carlos III, Valencia, Spain; 4 Respiratory Unit, University and Polytechnic La Fe Hospital, Valencia, Spain; 5 Almirall, R&D Centre, Barcelona, Spain; 6 Research and Teaching Unit, University General Hospital Consortium, Valencia, Spain

**Keywords:** Drug discovery, Biomarkers, Molecular medicine, Pathogenesis, Respiratory tract diseases

## Abstract

Non-T2 severe asthma and chronic obstructive pulmonary disease (COPD) are airway chronic inflammatory disorders with a poor response to corticosteroids. LAS194046, a novel pan-Janus kinase (JAK) inhibitor, shows inhibitory effects on T2 allergic lung inflammation in rats. In this work we analyze the effects of LAS194046, fluticasone propionate and their combination in neutrophils from non-T2 severe asthma and COPD patients in vitro. Neutrophils from 23 healthy subjects, 23 COPD and 21 non-T2 severe asthma patients were incubated with LAS194046 (0.01 nM–1 µM), fluticasone propionate (0.1 nM–1 µM) or their combination and stimulated with lipopolysaccharide (LPS 1 µM). LAS194046 shows similar maximal % inhibition and potency inhibiting IL-8, MMP-9 and superoxide anion release in neutrophils from healthy, COPD and asthma. Fluticasone propionate suppresses mediator release only in neutrophils from healthy patients. The combination of LAS194046 with fluticasone propionate shows synergistic anti-inflammatory and anti-oxidant effects. The mechanisms involved in the synergistic effects of this combination include the increase of MKP1 expression, decrease of PI3Kδ, the induction of glucocorticoid response element and the decrease of ERK1/2, P38 and JAK2/STAT3 phosphorylation compared with monotherapies. In summary, LAS194046 shows anti-inflammatory effects in neutrophils from COPD and severe non-T2 asthma patients and induces synergistic anti-inflammatory effects when combined with fluticasone propionate.

## Introduction

Chronic airway inflammation is a key feature in severe asthma and chronic obstructive pulmonary disease (COPD), which is poorly controlled by inhaled or systemic corticosteroids^1^. Searching for new anti-inflammatory therapies to improve chronic inflammation and to improve the anti-inflammatory effects of corticosteroids has been a constant in the last decades of asthma and COPD pharmacologic research. In this regard, the first introduction of anti-leukotrienes, improved the inflammatory profile of mild allergic asthma, but not in severe asthma. The recent introduction of biologic treatments, such as the anti-IgE, anti-IL-5 and anti-IL-4Rα (IL-4/IL-13) monoclonal antibodies have change the management of severe asthma. However, biologic treatments show efficacy in T2 eosinophilic severe asthma (> 300 eosinophils/µL) patients^2^, improving inflammatory profile, lung function, symptoms and quality of life, although the long term clinical remission on treatment is reached only in 14–28% of patients with this asthma phenotype^3^ In contrast, non-T2 inflammatory asthma phenotypes, represented by mixed or pure forms of T1/T17 or neutrophilic inflammation shows less response to inhaled/systemic corticosteroids and biologic treatments, representing around 30% of severe asthma phenotypes with a poor prognosis^4,5^. Neutrophilic airway inflammation is present in individuals who had generally adult-onset and severely obstructed (and incompletely reversible) asthma and the highest-intensity healthcare usage and systemic corticosteroid use^6^ which shares similarities with COPD (namely asthma-COPD overlap). Similar immunobiology profile has been observed in mild-severe COPD patients showing increased number and activated neutrophils in sputum and blood, characterized by an elevated oxidative stress, matrix metalloproteinases, and increased number of cytokines and chemokines such as IL-8 and TNFα between many others^7^. Knowing that many patients with severe asthma and COPD suffer with persistent symptoms despite the use of high doses of inhaled corticosteroids (ICS) and that new biologic treatments are limited to T2 eosinophilic severe asthma and not useful for COPD, novel drugs that target inflammation may be of therapeutic benefit.

Most pro-inflammatory cytokines driving severe asthma and COPD signal through Janus Kinase (JAK) proteins following binding to cytokine receptors, resulting in activation of signal transducer and activator of transcription (STAT) proteins^8^. JAK family of cytoplasmic tyrosine kinases comprises four isoforms, JAK1, JAK2, JAK3, and Tyrosine kinase 2 (TYK2). The active phosphorylated forms of JAK activate and phosphorylate STAT proteins which dimerize and move to the nucleus to activate gene expression involved in inflammatory responses^9^.

Relevant pro-inflammatory cytokines associated with innate immune response such as type II interferon (IFN)γ, IL-6, IL-5, IL-3, IL-12, IL-13 and IL23 receptors interact with JAK1, JAK2 and TYK2, while IL-2, IL-4, IL-7, IL-9, IL-15 and IL-21 receptors preferentially interact with JAK3 and JAK1 and are primarily associated with adaptive immunity^10^.

Current pan-JAK inhibitors such as baricitinib and tofacitinib are approved for the treatment of inflammatory diseases such as rheumatoid arthritis, and in development for ulcerative colitis and Crohn’s disease. In lung diseases, pan-JAK inhibitors have shown promising results in in vitro models with inflammatory cells isolated from asthma patients, where the pan-JAK inhibitor tofacitinib reduced cytokine levels and showed additive effect in combination with corticosteroids inhibiting lymphocytes^11^. In animal models of airway inflammation, pan-JAK inhibitors improved lung inflammation^12–15^ suggesting a favorable profile in lung inflammatory diseases such as asthma and COPD. However, to our knowledge, there is no data on the effect of pan-JAK inhibitors on neutrophil activation.

The aim of this study was to evaluate the anti-inflammatory profile of a pan-JAK inhibitor, LAS194046, on human neutrophils from severe asthma and COPD patients in vitro, as well as to analyze possible additive effects improving anti-inflammatory effects of corticosteroids. Results obtained in this study may be of potential value to understand the effects of the JAK/STAT pathway inhibition on neutrophil inflammation in airway diseases such as non-T2 severe asthma and COPD.

## Materials and methods

All reagents were obtained from Sigma-Aldrich (St. Louis, MO, USA) unless otherwise stated. All experiments were performed in accordance with relevant named guidelines and regulations.

### Patients

Sputum neutrophils and peripheral blood neutrophils were obtained from healthy subjects, severe COPD and severe asthmatic patients at the University General Hospital of Valencia and Hospital La Fe of Valencia, Spain. Pulmonary function tests (forced spirometry) and arterial blood gas measurements were performed during the days prior to sampling. According to their spirometry results and smoking habits, patients were classified into three groups: (A) Healthy subjects, patients with normal lung function and who did not smoke; (B) Severe COPD patients “D” as described in the Global Initiative for Obstructive Lung Disease guidelines (GOLD, 2015): frequent exacerbators (≥ 2/year), highly symptomatic (dyspnea scale mMRC ≥ 2 or COPD assessment test CAT ≥ 10) and airflow limitation 3–4; (C) Severe asthmatics as described in the Global Initiative for Asthma (GINA, 2015) guidelines: uncontrolled patients despite high dose of iCS/LABA in Step 4 and 5 of management.

Hereafter, when we indicate asthma or COPD, we refer to the severe forms of these diseases. 21 patients with asthma and 23 patients with COPD, were enrolled in this study. COPD patients were aged 65.5 ± 11 years, FEV1 32.1 ± 9% predicted, and 19 were prescribed an inhaled corticosteroid. All COPD patients were current smokers. Asthma patients were aged 55 ± 10 years, all of them were prescribed an inhaled corticosteroid, and only 6 were prescribed oral steroids. In both cases, there were no exacerbations of the disease within 2 weeks prior to taking blood samples.

23 age-matched non-smoking control subjects with normal lung function (age 66 ± 6 years old, FEV1 99 ± 6% predicted) who did not have any respiratory disease, were also recruited as normal controls, respectively. Routine lung function tests were performed to evaluate forced vital capacity (FVC), forced expiratory volume in 1 s (FEV1) and FEV1/FVC ratio using a spirometer (Vitalograph, Maids Moreton, UK). The clinical features of the study population are summarised in Supplementary File 1 (Table S1 and Table S2). This project was approved by the local ethics committee of General University Hospital and Hospital La Fe, Valencia, Spain, and written informed consent was taken from each patient or volunteer before starting blood sampling.

### Human neutrophil isolation from peripheral blood and sputum

Neutrophils were isolated from peripheral venous blood of asthma and COPD patients, and cultured as previously outlined^16^. Using 3% dextran 500 (in 0.9% saline) together with Ficoll-Paque Histopaque 1077 (Amersham Pharmacia Biotech, Barcelona, Spain) at a ratio of 2:1. The neutrophil preparations were > 97% pure as assessed by Giemsa staining and had viability of > 99% as measured by trypan blue exclusion. Neutrophils from spontaneous sputum (~ 2 ml) were collected from patients with COPD and processed with dithiothreitol using established methods^17^. Sputum cell pellets were resuspended in RPMI 1640 supplemented with 10% foetal calf serum, 1% penicillin–streptomycin and 1 mmol l-glutamine/l at a concentration of 1 × 10^6^ cells/ml. An aliquot containing 4 × 10^5^ cells was incubated on a 24-well plate for 1 h at 37 °C in humidified 5% CO_2_. Preparations containing < 95% neutrophils were discarded. Neither the purity nor the viability of the cell preparations was affected by the different experimental conditions of the study.

### Preparation of cigarette smoke extract solutions

The smoke of a research cigarette (2R4F; Tobacco Health Research, University of Kentucky, Lexington, KY, USA) was generated by a respiratory pump (Rodent Respirator 680; Harvard Apparatus, March-Hugstetten, Germany) through a puffing mechanism mimicking the human smoking pattern (3 puffs/min; 1 puff 35 ml; each puff of 2 s duration, 0.5 cm above the filter) and was bubbled into a flask containing 25 ml of pre-warmed (37 °C) RPMI 1640 culture medium. The resulting CSE solution was considered as 100% CSE and used for experiments within 30 min of preparation. CSE 10% corresponded approximately to the exposure associated with smoking two packs of cigarettes per day^18^. To test for cytotoxicity/apoptosis induced by CSE, isolated neutrophils were treated with CSE concentrations of up to 5% for 6 h. No significant difference in the lactate dehydrogenase level (lactate dehydrogenase cytotoxicity assay; Cayman Chemical, Madrid, Spain) or annexin V-FITC was observed between the CSE and control groups.

### Cell stimulations, IL-8 and MMP-9 assays

Sputum and peripheral blood neutrophils were adjusted to 500 × 10^3^ cells per well in 24-well plates and incubated in RPMI 1640 for 1 h at 37 °C, 5% CO_2_. The cells were then left untreated or treated with the pan-JAK inhibitor LAS194046 (0.01 nM–10 µM; Almirall SA, Barcelona, Spain), or the corticosteroid fluticasone propionate (0.1 nM–1 µM), as well as their combinations for 1 h before cells were stimulated with 1 µg/ml of lipopolysaccharide (LPS) from *Salmonella* *enterica* (cat no. L7770), CSE 5% or 1 µM *N*-formyl methionine-leucyl-phenylalanine (fMLP) (cat no. F3506) at the indicated times. Drugs were dissolved in dimethyl sulfoxide (DMSO) at 10 mM stock concentrations. Several dilutions of the stocks were performed with cell culture medium. The final concentrations of DMSO (0.1%) in the cell culture did not affect cellular functions.

CSE 5% was selected as a stimulus in sputum neutrophils because LPS alone did not increase interleukin (IL)-8 levels over basal values, as previously reported^19^.

After the stimulation period, supernatants were collected and centrifuged at 120×*g* for 5 min. The cell-free supernatant was used to measure IL-8 and metalloproteinase-9 (MMP9). Cellular extracts were used to measure mRNA expression after 6 h of cell stimulation. IL-8 and MMP-9 levels were measured using a commercially available enzyme-linked immunosorbent assay kits for IL-8 and MMP-9 (R&D Systems, Nottingham, UK) according to the manufacturer’s protocol.

### Reactive oxygen species generation by human neutrophils

Isolated neutrophils were placed in black culture 96-well plates in RPMI-1640 culture medium, and pre-incubated with LAS194046 (0.01 nM–10 µM), fluticasone propionate (0.01 nM–10 µM) and their combinations for 30 min, and then stimulated with fMLP 1 µM between 0 to 45 min. The fMLP stimulus was selected for superoxide anion formation because its robust induction on the reactive oxygen species that is reproducible in different models of pharmacology testing^16,20,21^.

Superoxide anions were determined based on chemiluminescence from luminol with the Superoxide Anion Assay Kit (Sigma Aldrich cat. no. CS1000) following the instructions of the manufacturer. In brief, neutrophils were incubated with different drugs, assay buffer, luminol solution and enhancer solution as specified by manufacturer, during 30 min, followed by the stimulation with fMLP, In presence of an “enhancer” superoxide anions convert luminol into a reactive peroxide that in several transitions emits energy as a photon producing a blue glow (chemiluminescence) that has been tested during 4 h as indicate manual instructions. Chemiluminescence was measured each 5 min, and Area Under Curve (AUC) were calculated for all time-dependent curves of superoxide anion during 45 min of fMLP stimulation.

### Real-time RT-PCR

Total RNA and reverse transcriptions were performed as previously reported^22^. cDNA was amplified using specific primers together with probes predesigned by Applied Biosystems for MKP-1 (cat. no. Hs00610256), PI3K-δ (cat. no. Hs00192399), GRα (cat. no. Hs00353740_m1), JAK1 (Hs01026983_m1), JAK2 (Hs01078136_m1), JAK3 (Hs01006616_m1), TYK2 (Hs01105965_m1), CD200 (Hs01033302_m1) genes in a 7900HT Fast Real-Time PCR system (Applied Biosystems) using Universal Master Mix (Applied Biosystems). Expression of the target gene was reported as the fold increase or decrease relative to the expression of GAPDH as an endogenous control (Applied Biosystems; 4310884E). The mean value of the replicates for each sample was calculated and expressed as the cycle threshold (Ct). The level of gene expression was then calculated as the difference (ΔCt) between the Ct value of the target gene and the Ct value of GAPDH. The fold changes in the target gene mRNA levels were expressed as 2^−ΔCt^.

### Glucocorticoid response element transfection assay

The Cignal Glucocorticoid Response Element (GRE) reporter assay kit (Qiagen, cat. no. 336841) was used to monitor the activity of glucocorticoid receptor-induced signal transduction pathways in cultured Beas2B bronchial epithelial cells as previously described in detail^22^.

### Western blot

Western blot analysis was used to detect changes in p-ERK1/2, p-P38, p-JAK2 and p-STAT3 using a rabbit anti-human p-ERK1/2 (1:1,000) antibody (monoclonal antibody; Cell Signaling, Boston, MA, USA; cat. no. 4376S) normalised to total rabbit anti-human ERK1/2 (1:1,000) antibody (monoclonal antibody; Cell Signaling; cat. no. 4695); rabbit anti-human phospho-P38 (1:1,000) antibody (monoclonal antibody; Cell Signaling; cat. no. 4631) normalised to total rabbit anti-human P38 (1:1,000) antibody (monoclonal antibody; Cell Signaling; cat. no. 9212), rabbit anti-human/rat phospho(p)-JAK2 (1:1000) antibody (monoclonal antibody; Novus Biologicals, Abingdon Oxon, UK; catalog no. NB110-57144) normalized to total rabbit anti-human/rat JAK2 (1:1000) antibody (polyclonal antibody; Novus Biologicals, Abingdon Oxon, UK; catalog no. NBP1-61916); rabbit anti-human/rat phospho(p)-STAT3 (1:1000) antibody (monoclonal antibody; Novus Biologicals, Abingdon Oxon, UK; catalog no. NB100-851) normalized to total rabbit anti-human/rat STAT3 (1:1000) antibody (polyclonal antibody; Novus Biologicals, Abingdon Oxon, UK; catalog no. NB100-91973), as previously outlined in detail^22^. The enhanced chemiluminescence method of protein detection using enhanced chemiluminescence reagents (ECL Plus; Amersham GE Healthcare, Buckinghamshire, UK) was used to detect labeled proteins. Densitometry of films was performed using the Image J 1.42q software (available at http://rsb.info.nih.gov/ij/, USA). Results of target protein expression are expressed as x-fold of the control condition and normalized to non-phosphorylated protein.

### PI3Kδ activity

To measure PI3Kδ activity, neutrophils from asthma and COPD patients were isolated and then incubated with LAS194046 (0.01 nM and 1 nM) or LY294002 (1 μM) for 1 h. The cells were stimulated with LPS for 30 min and then centrifuged. Total protein was extracted and PI3Kδ activity was measured as described in detail previously^22^.

### Analysis of results

The data were subjected to a parametric analysis, with p < 0.05 considered indicative of statistical significance. Data are expressed as the mean ± SD of n experiments using a Student’s *t* test and one-way or two-way analysis of variance (ANOVA) followed by a Bonferroni post hoc test, using the GraphPad v.6 software.

### Ethics approval and consent to participate

This project was approved by the local ethics committee of General University Hospital and Hospital La Fe, Valencia, Spain, and written informed consent was taken from each patient or volunteer before starting blood sampling. All experiments were performed in accordance with relevant named guidelines and regulations.

## Results

### Anti-inflammatory and antioxidant effects of LAS194046 and fluticasone propionate in neutrophils from healthy and COPD patients

**Figure 1:**
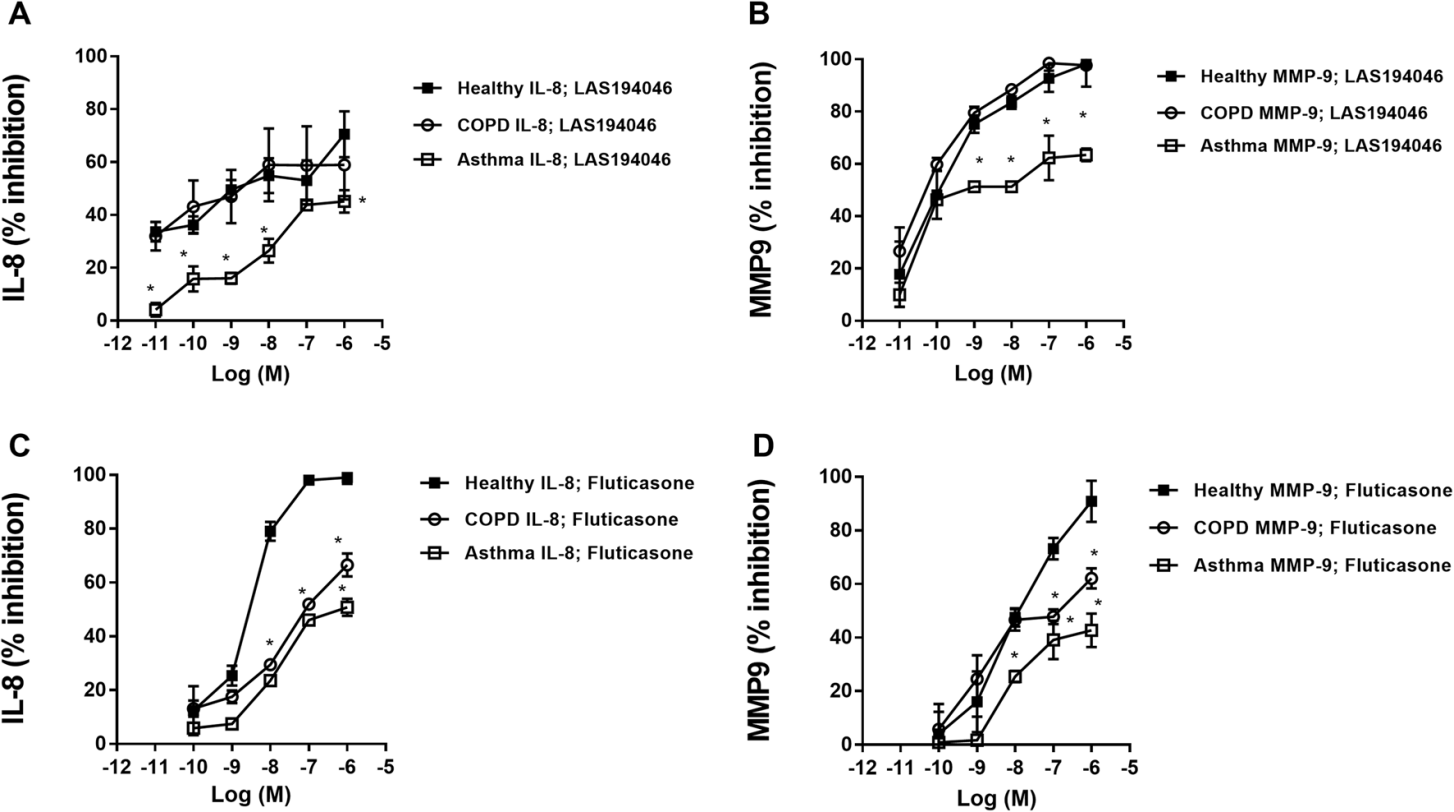
Effects of LAS194046 and fluticasone propionate on human neutrophil inflammation. Concentration-dependent inhibition of lipopolysaccharide (LPS)-induced IL-8 or MMP-9 release by (**A**,**B**) LAS194046 and (**C**,**D**) fluticasone propionate from peripheral blood neutrophils of healthy controls, severe asthma and COPD patients. Neutrophils were pre-incubated with LAS194046 or fluticasone propionate during 1 h followed by cell stimulation with LPS (1 µg/ml) for 6 h. The results are expressed as the mean ± SD (n = 3 each for cells from healthy controls, severe asthma and COPD patients in independent experiments with triplicate samples). A two-way ANOVA was followed by a post hoc Bonferroni test. *p < 0.05 vs. cells from healthy patients.

**Figure 2:**
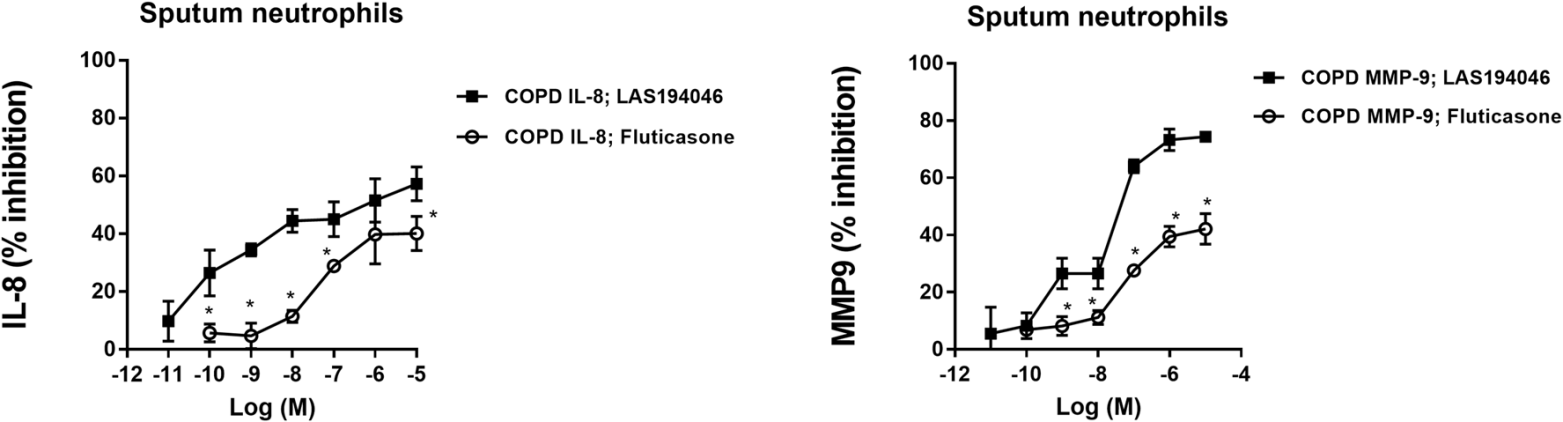
Effects of LAS194046 and fluticasone propionate on COPD sputum human neutrophil inflammation. Concentration-dependent inhibition of cigarette smoke extract (CSE)-induced (**A**) IL-8 or (**B**) MMP-9 release by LAS194046 and fluticasone propionate from sputum neutrophils of COPD patients. Neutrophils were pre-incubated with LAS194046 or fluticasone propionate during 1 h followed by cell stimulation with CSE (5%) for 6 h. The results are expressed as the mean ± SD (n = 3 each for cells from COPD patients in independent experiments with triplicate samples). A two-way ANOVA was followed by a post hoc Bonferroni test. *p < 0.05 vs. cells from fluticasone propionate.

LAS194046 concentration-dependently (0.01 nM–1 μM) inhibited the mediator secretion induced by LPS (1 µg/ml) similarly in both healthy and COPD neutrophils, showing the maximal % inhibition of 70.54 ± 15% and 58.95 ± 22.64% for IL-8 respectively, and 98.1 ± 14.86% and 97.68 ± 3.29% for MMP9 release (Fig. 1A,B, Supplementary File 1: Table S3). The maximal % inhibition in neutrophils from severe asthma patients was 45.1 ± 7.5% and 63.44 ± 2.44 for IL-8 and MMP9 release, significantly lower than in neutrophils from healthy and COPD patients (Fig. 1A,B Supplementary File 1: Table S3). Fluticasone propionate showed significant less maximal % inhibition in neutrophils from severe asthma and COPD patients than in healthy neutrophils. Thus, the maximal % inhibition of fluticasone propionate was 99.01 ± 2.26, 66.57 ± 7.45 and 50.77 ± 5.43 for IL-8 release in healthy, COPD and asthma neutrophils respectively, and 90.85 ± 7.66, 62.09 ± 3.79 and 42.72 ± 6.25 for MMP9 in healthy, COPD and asthma neutrophils respectively (Fig. 1C,D, Supplementary File 1: Table S3). In sputum neutrophils from COPD patients, the maximal % inhibition of LAS194046 was 57.27 ± 10.13% and 74.34 ± 3.09 for IL-8 and MMP9 release, significantly higher than maximal % inhibition of fluticasone propionate which reached 40.12 ± 10.30% and 42.14 ± 9.23% inhibition for IL-8 and MMP9 respectively (Fig. 2, Supplementary File 1: Table S3).

**Figure 3:**
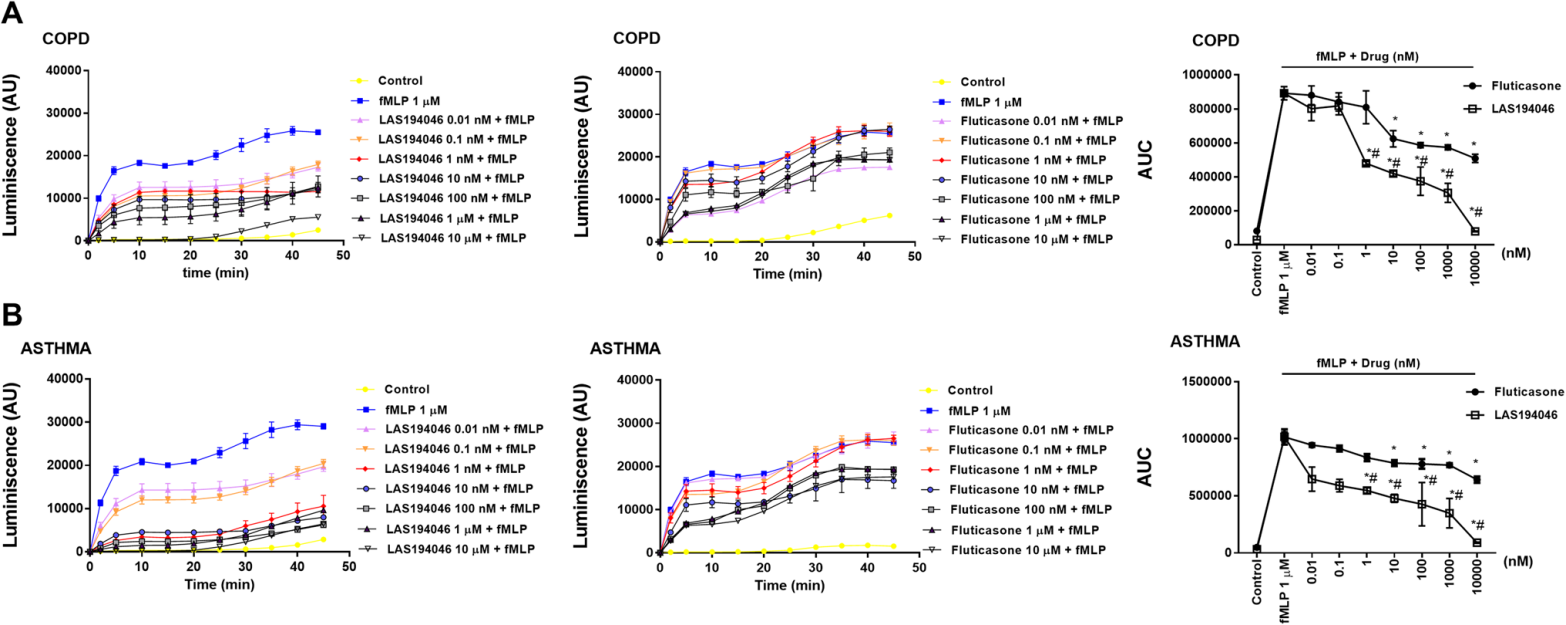
Effects of LAS194046 and fluticasone propionate on human neutrophil superoxide anion formation. Concentration-dependent inhibition of *N*-Formylmethionyl-leucyl-phenylalanine (fMLP)-induced superoxide anion formation by LAS194046 and fluticasone propionate from peripheral blood neutrophils of (**A**) COPD and (**B**) severe asthma patients. Neutrophils were pre-incubated with LAS194046 or fluticasone propionate during 30 min followed by cell stimulation with fMLP (1 µM) for up to 45 min. The results are expressed as luminescence values [arbitrary units (AU)] and as area under curve generated by luminescence time-curse data as the mean ± SD (n = 3 each for cells from COPD patients in independent experiments with triplicate samples). A two-way ANOVA was followed by a post hoc Bonferroni test. *p < 0.05 vs. control; ^#^p < 0.05 vs. fluticasone propionate.

fMLP 1 µM induced the increase of superoxide anion formation in neutrophils from severe asthma and COPD that was concentration-dependently (0.01 nM–10 μM) inhibited by LAS194046, reaching a maximal % inhibition of 98 ± 2% and 97.8 ± 4% respectively, significantly higher than the maximal % inhibition reached by fluticasone propionate that was 48.1 ± 4% and 45.4 ± 5% (Fig. 3A,B).

### LAS194046 and fluticasone propionate combination improves anti-inflammatory and anti-oxidant effects in neutrophils from severe asthma and COPD patients

**Figure 4:**
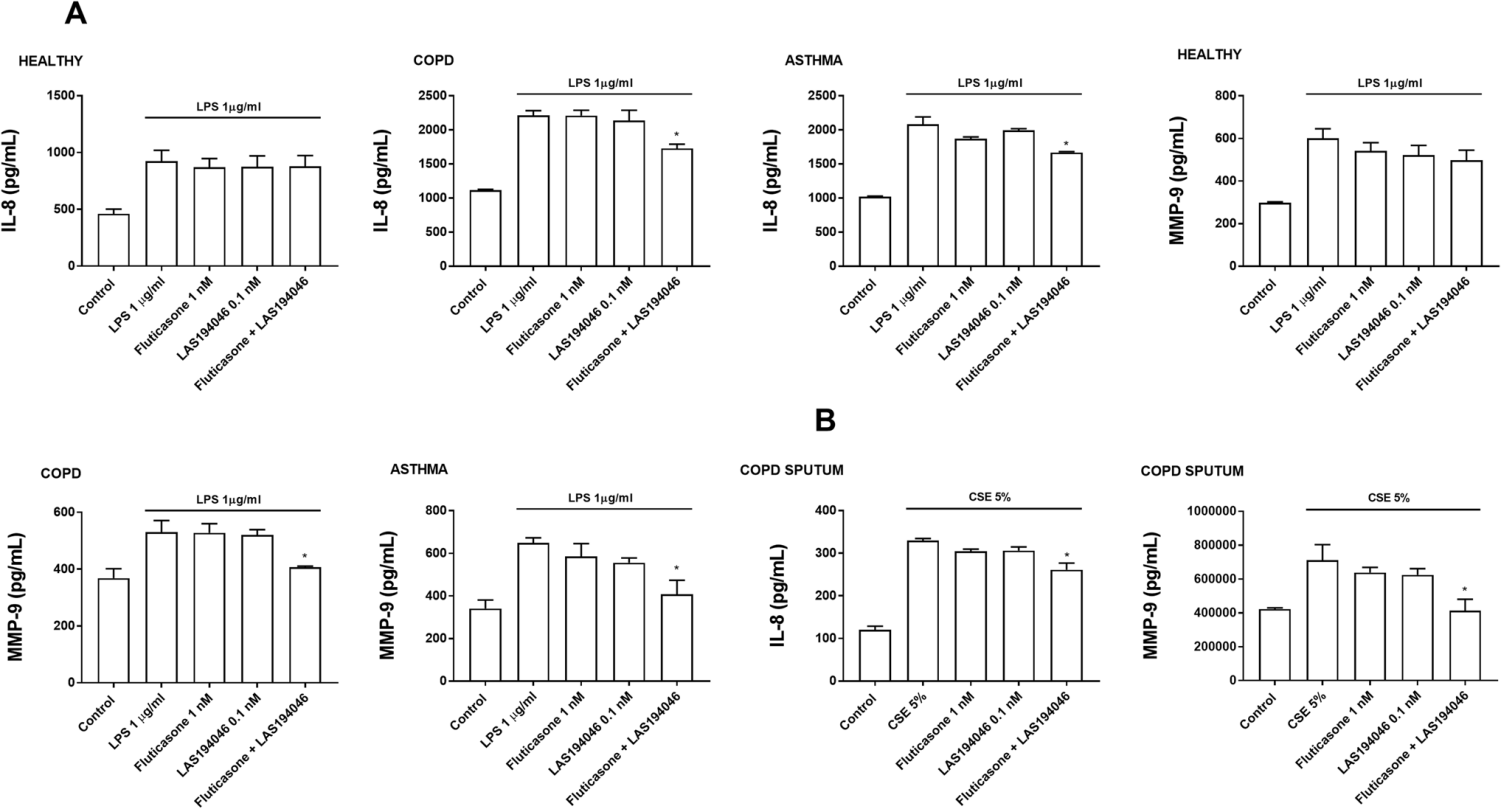
LAS194046 and fluticasone propionate combination shows additive/synergic effects on human neutrophil inflammation. Neutrophils from healthy subjects, severe asthma and COPD patients were pre-incubated with sub-effective concentrations of LAS194046, fluticasone propionate or its combination during 1 h followed by cell stimulation with (**A**) LPS (1 µg/ml) or (**B**) cigarette smoke extract (CSE 5%) for 6 h to measure IL-8 and MMP-9 release. The results are expressed as the mean ± SD (n = 3 each for cells from healthy controls, severe asthma and COPD patients in independent experiments with triplicate samples). A two-way ANOVA was followed by a post hoc Bonferroni test. *p < 0.05 vs. cells from healthy patients.

**Figure 5:**
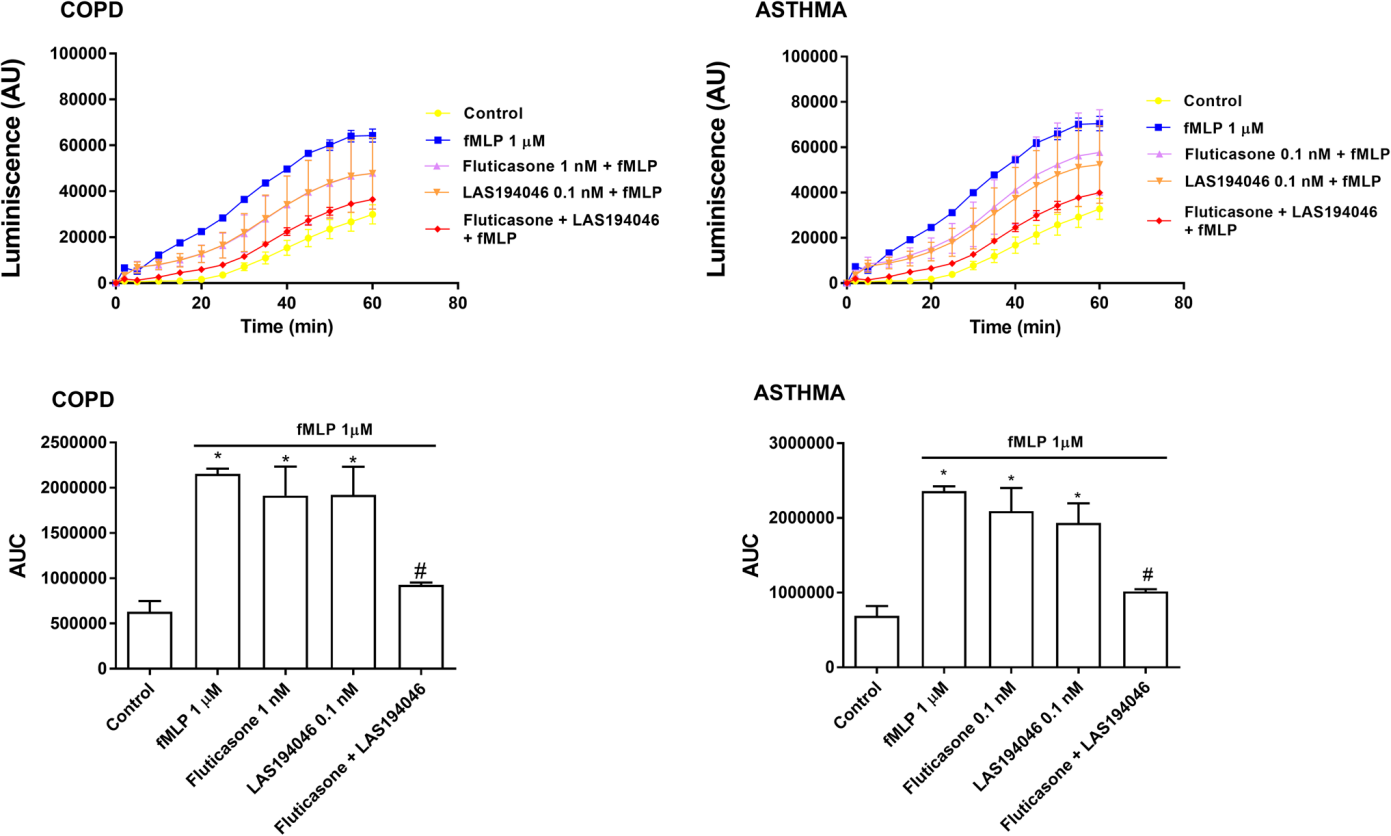
LAS194046 and fluticasone propionate combination shows additive/synergic effects on human neutrophil superoxide anion formation. Neutrophils from severe asthma and COPD patients were pre-incubated with sub-effective concentrations of LAS194046, fluticasone propionate or its combination during 30 min followed by cell stimulation with fMLP (1 µM) for 45 min. The results are expressed as luminescence values [arbitrary units (AU)] and as the area under curve generated by luminescence time-curse data as the mean ± SD (n = 3 each for cells from COPD patients in independent experiments with triplicate samples). A two-way ANOVA was followed by a post hoc Bonferroni test. *p < 0.05 vs. control; ^#^p < 0.05 vs. monotherapy.

Neutrophils were incubated with non-effective concentrations of LAS194046 0.1 nM, fluticasone propionate 1 nM or their combination 1 h before cells were stimulated with 1 µg/ml of LPS. While the monotherapy did not reach significant inhibition, the combination of LAS194046 0.1 nM and fluticasone propionate 1 nM reached significant inhibition of LPS-induced IL-8 and MPP9 release in peripheral blood neutrophils from severe asthma and COPD patients (Fig. 4A) as well as in neutrophils from sputum of COPD patients (Fig. 4B). The inhibitory effects of combination were not observed in neutrophils from healthy donors (Fig. 4A). Similar effects were observed on superoxide anion release. In this case, combination of the LAS194046 0.1 nM and fluticasone propionate 1 nM showed synergic effects inhibiting superoxide anion generation in neutrophils from COPD and severe asthma patients (Fig. 5).

### Expression of JAK isoforms and corticosteroid target genes in neutrophils from severe asthma and COPD

**Figure 6:**
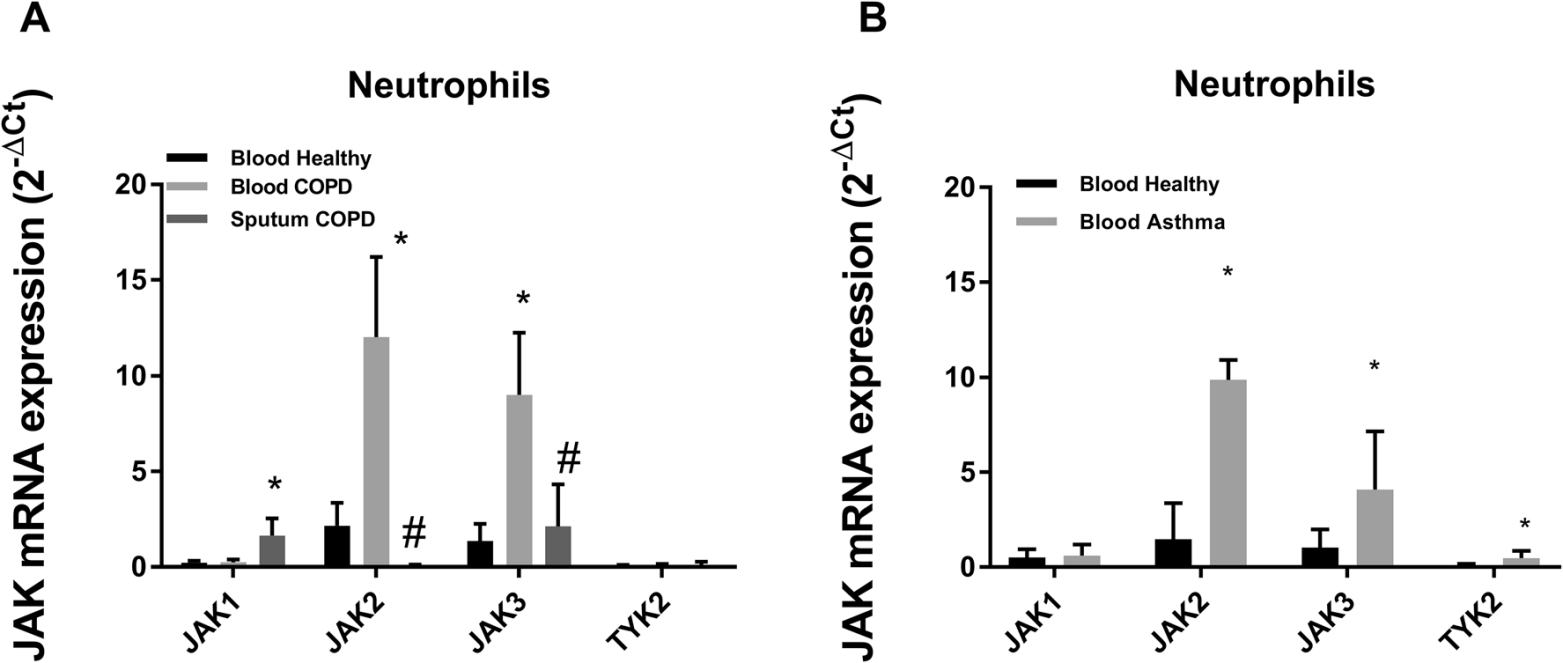
Basal JAK isoforms gene expression. Basal gene expression of JAK isoforms in peripheral blood neutrophils from healthy (n = 23), (**A**) COPD (n = 23) and (**B**) severe asthma patients (n = 21), and (**A**) from sputum COPD neutrophils (n = 22). Relative quantification was determined with the 2^−ΔCt^ method. Results are expressed as mean ± SD. *p < 0.05 vs. healthy: ^#^p < 0.05 vs. blood COPD neutrophils.

Neutrophils from healthy subjects, severe asthma and COPD patients showed a higher expression of JAK2 followed by JAK3 and in a lesser extent of JAK1, while levels of TYK2 were the lowest (Fig. 6A). Between different subjects, the expression of the different JAK isoforms was higher in blood neutrophils from COPD and severe asthma, followed by sputum neutrophils from COPD patients that were significantly higher than in blood neutrophils from healthy subjects (Fig. 6A,B).

**Figure 7:**
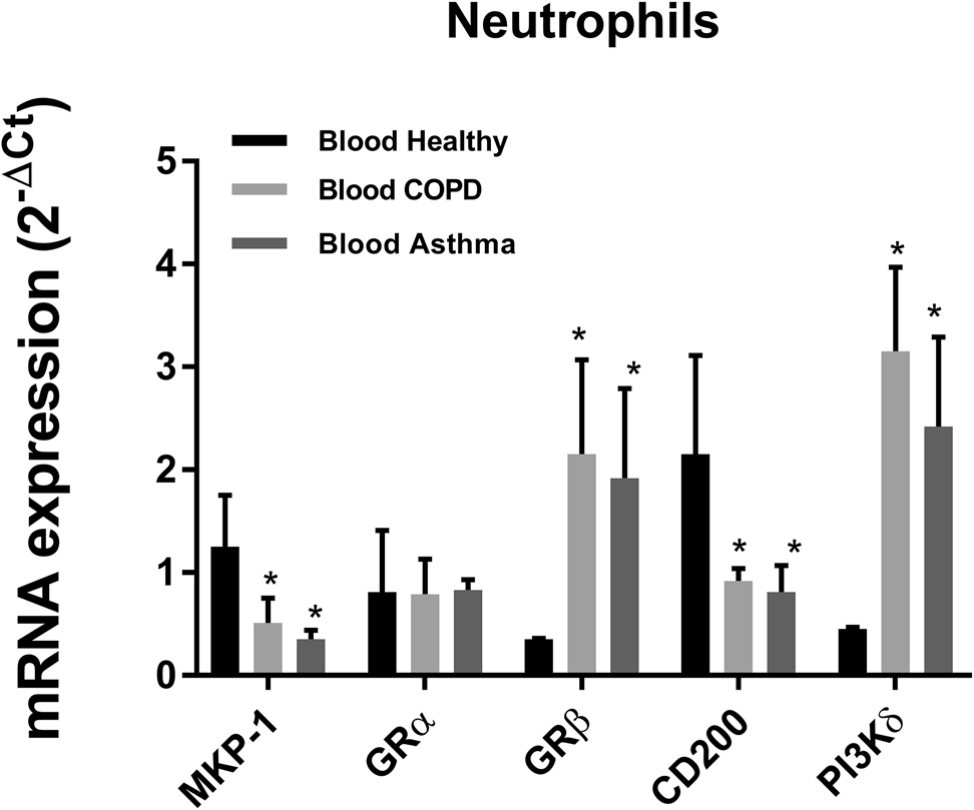
Basal corticosteroid dependent gene expression. Basal gene expression of corticosteroid anti-inflammatory genes (MKP-1, GRα and CD200) and corticosteroid inhibitor genes (PI3Kδ and GRβ) in peripheral blood neutrophils from healthy (n = 23), COPD (n = 23) and severe asthma patients (n = 21). Relative quantification was determined with the 2^−ΔCt^ method. Results are expressed as mean ± SD. *p < 0.05 vs. healthy.

The anti-inflammatory effects of corticosteroids are mediated, in part, by the induction of anti-inflammatory genes such as MKP-1 and CD200 between others, following the interaction with the intracellular receptor GRα^23^. The expression of CD-200 and MKP-1 were downregulated in neutrophils from severe asthma and COPD patients while GRα was not modified (Fig. 7). The expression of non-functional GRβ and the corticosteroid negative modulator PI3Kδ were increased in neutrophils from severe asthma and COPD patients (Fig. 7).

### Mechanisms of additive/synergic effects between LAS194046 and fluticasone propionate

**Figure 8:**
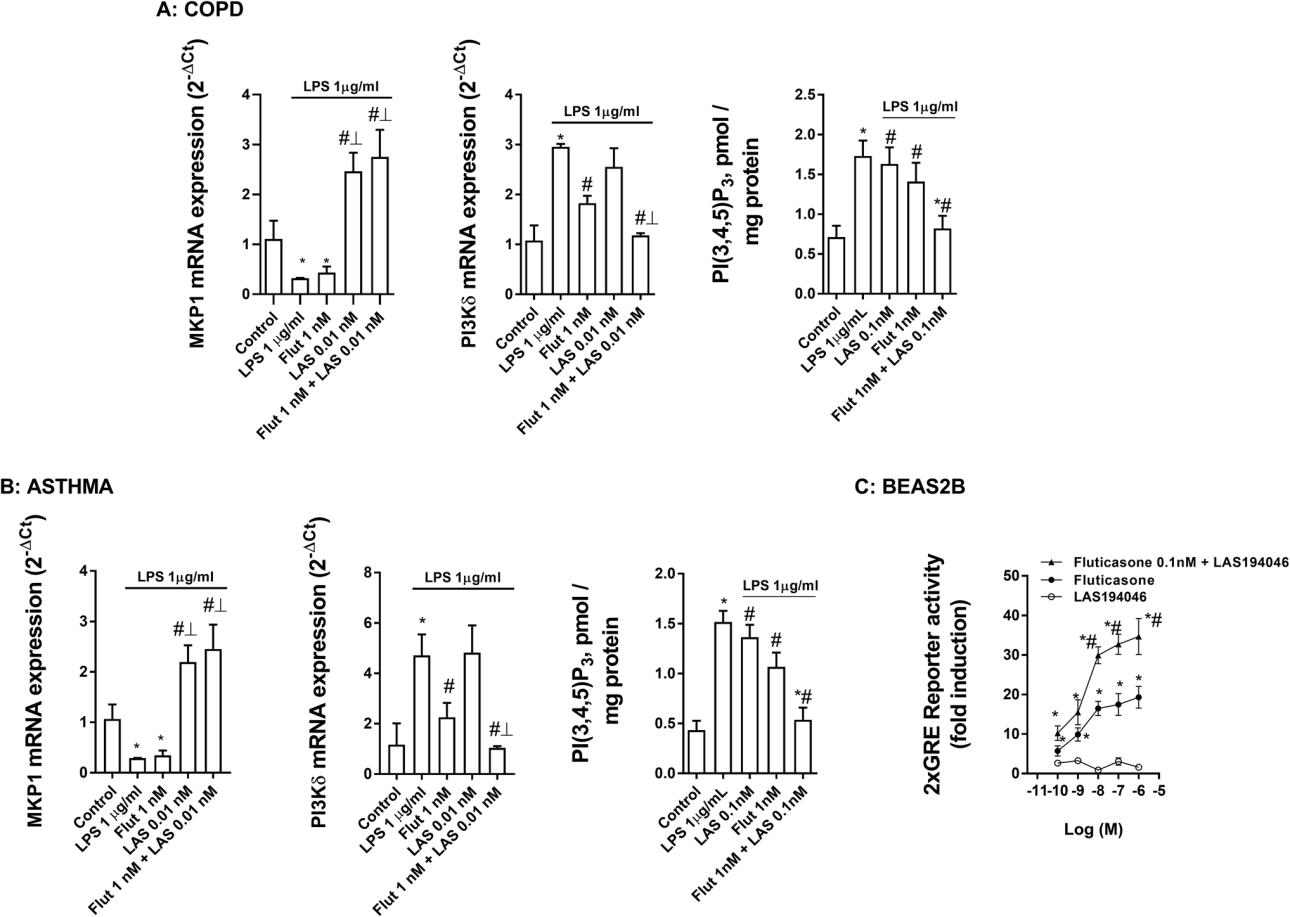
Mechanistic effects of LAS194046 improving corticosteroids anti-inflammatory effects. Neutrophils from (**A**) COPD and (**B**) severe asthma patients were pre-incubated with sub-effective concentrations of LAS194046, fluticasone propionate or its combination during 1 h followed by cell stimulation with LPS (1 µg/ml) for 6 h. MKP1 and PI3Kδ gene expression and PI3Kδ activity were measured. The results are expressed as the mean ± SD (n = 3 each for cells from severe asthma and COPD patients in independent experiments with triplicate samples). A two-way ANOVA was followed by a post hoc Bonferroni test. *p < 0.05 vs. control; ^#^p < 0.05 vs LPS. (**C**) Bronchial epithelial Beas2B cells were transfected with a GRE reporter gene and stimulated with different combinations of LAS194046 and fluticasone propionate. The results are expressed as the mean ± SD (n = 4 run in triplicate). A one-way ANOVA was followed by a post hoc Bonferroni test. *p < 0.05 vs. LAS194046 monotherapy; ^#^p < 0.05 vs. fluticasone propionate monotherapy.

**Figure 9:**
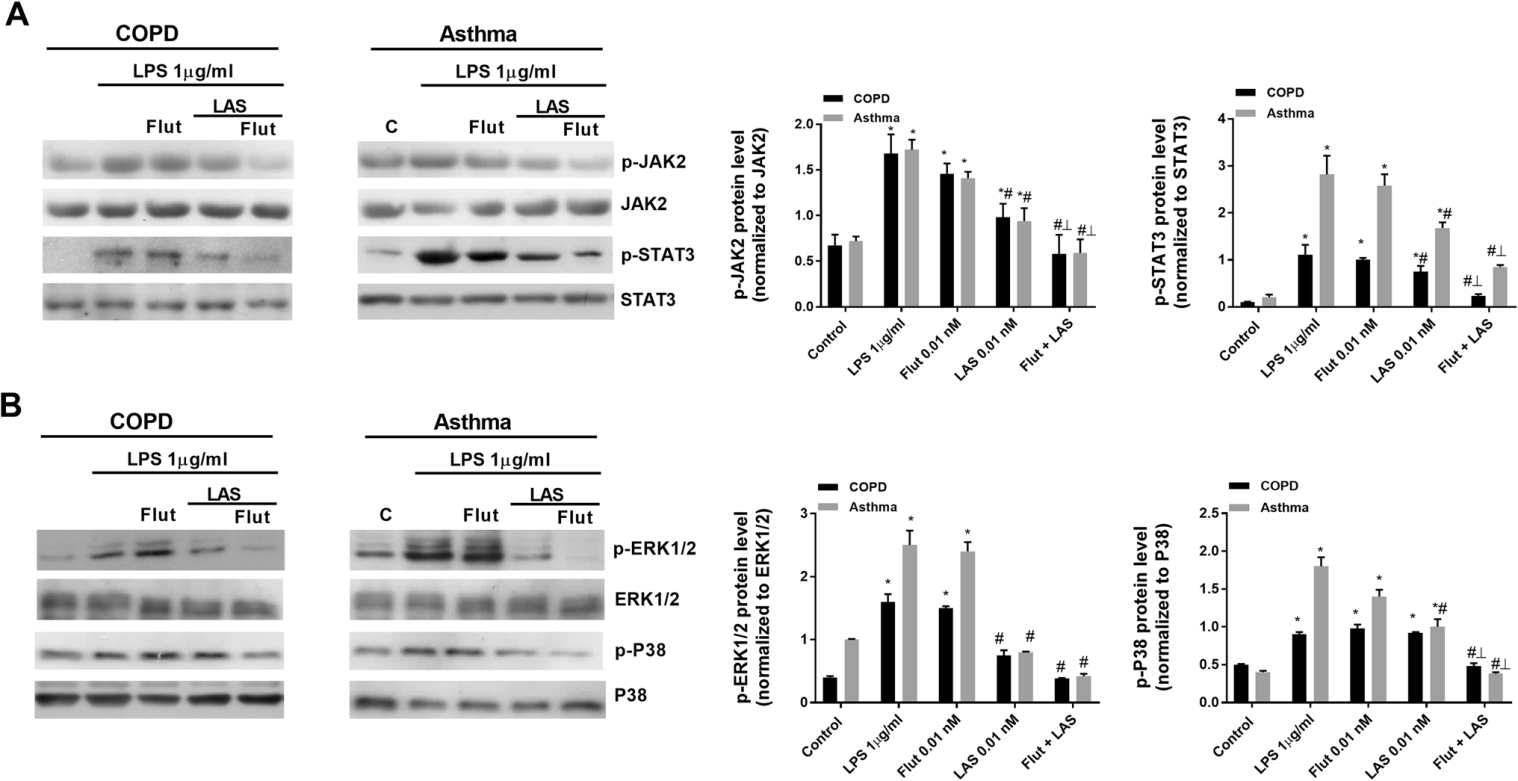
LAS194046 and fluticasone propionate combination shows additive/synergic effects inhibiting the phosphorylation of JAK2/STAT3 and ERK1/2-P38 pathways in neutrophils from severe asthma and COPD patients. Neutrophils from severe asthma and COPD patients were pre-incubated with sub-effective concentrations of LAS194046, fluticasone propionate or its combination during 1 h followed by cell stimulation with LPS (1 µg/ml) for 1 h to measure the phosphorylation of (**A**) JAK2/STAT3 and (**B**) ERK1/2-P38 by western blot. Extended blots are presented in Supplementary Figs. S1 and S2 online. The results are expressed as the mean ± SD (n = 3 each for cells from severe asthma and COPD patients). A one-way ANOVA was followed by a post hoc Bonferroni test. *p < 0.05 vs. control; ^#^p < 0.05 vs fluticasone propionate monotherapy; ^┴^p < 0.05 vs monotherapy.

The stimulation of neutrophils with LPS decreased the expression of MKP-1 and increased the expression and activity of PI3Kδ which was reversed by LAS194046 at sub-effective concentrations of 0.01 nM. The combination of LAS194046 0.01 nM and fluticasone propionate 0.1 nM showed additive effects increasing MKP-1 and inhibiting PI3Kδ expression and activity. These results were reproducible in neutrophils from peripheral blood of severe asthma and COPD patients (Fig. 8A,B). In other experiments performed in beas2b bronchial epithelial cells, transfected with GRE reporter gene, the combination of a fixed concentration of 0.1 nM of fluticasone with growing concentrations of LAS194046 potentiated the activation of the glucocorticoid response element (GRE), demonstrating the molecular potentiation of the anti-inflammatory effects of corticosteroids when the pan-JAK inhibitor LAS194046 is added (Fig. 8C). The LPS stimulation of human neutrophils from severe asthma and COPD patients increased the phosphorylation of JAK2 and STAT3 as well as the phosphorylation of ERK1/2 and P38. The non-effective concentration of fluticasone propionate 0.01 nM combined with LAS194046 potentiates the inhibitory effect of both drugs, suppressing the phosphorylation of JAK2/STAT3, ERK1/2 and P38 (Fig. 9A,B).

## Discussion

The aim of this study was to generate in vitro evidence that JAK/STAT pathway inhibition reduces neutrophil activation from patients with severe asthma and COPD and improves the anti-inflammatory effects of corticosteroids. We showed novel evidence on the inhibitory effects of LAS194046, a recent developed pan-JAK inhibitor, on activated neutrophils from severe asthma and COPD patients, reducing IL-8 and MMP9 secretion and superoxide anion generation, as well as improving the impaired anti-inflammatory effects of corticosteroids in neutrophils from severe asthma and COPD patients. The mechanisms of the additive/synergic effects of the LAS194046 and fluticasone propionate combination included the activation of the GRE gene sequence, the induction of MKP1, the inhibition of JAK2/STAT3, P38 and ERK1/2 phosphorylation, as well as the reduction of PI3Kδ expression and activity. These data represent novel information about the pan-JAK inhibitor, LAS194046, which previously showed inhibitory effects on ovoalbumin-induced T2 allergic lung inflammation in rats^14^.

Neutrophil inflammation is commonly observed in airways from COPD and severe asthma patients with non-T2 inflammation. The activation of peripheral and lung neutrophils causes the release of a number of mediators, including neutrophil elastase, MMP-9, IL-8, IL-1β and reactive oxygen species (ROS) that induce lung structural changes, including mucus secretion, bronchial smooth muscle hypertrophy and fibrosis in asthma and COPD, and emphysema in COPD, characterized by loss of respiratory bronchioles, alveolar ducts, and alveoli^7^ contributing to bronchial obstruction and irreversibility.

In addition, neutrophils from severe asthma and COPD are primed to respond exaggeratedly to different stimulus when compared with neutrophils from mild asthma and healthy donors^24,25^. However it appears that neutrophil priming/activation is more dependent on asthma severity than T2/non-T2 inflammatory phenotype. In this regard it has been shown that neutrophils from aspirin-exacerbated respiratory disease (AERD), a severe form of adult-onset eosinophilic asthma, shows a primed status to release large amounts of ROS, MMP-9 and neutrophil elastase in a similar way than in severe non-T2 asthma^26^. Therefore, the difference between T2 and non-T2 neutrophilic asthma inflammation appears to be the increased number of neutrophils, and neutrophil chemoattractants such as IL-17 or IL-8 in non-T2 inflammation. In this regard, increased number of neutrophils in asthma correlates with persistent airflow limitation and asthma severity^27,28^, showing and inflammatory phenotype resistant to corticosteroids, which suggest neutrophils as immunotherapeutic target for severe asthma^29^. Currently there is no evidence on whether there are different effects of LAS194046 in neutrophils from T2-asthma and non-T2 asthma which guarantees future studies.

Bronchial chronic bacterial colonization is typically observed in non-T2 severe asthma and COPD patients, including gram-negative bacteria expressing LPS^7,30^, which activates toll like receptor 4 to promote inflammatory responses of the innate immunity.

In this work, inflammatory LPS or an oxidative CSE stimulus activated human neutrophils to release IL-8 and MMP9 that was effectively inhibited by the corticosteroid fluticasone propionate in neutrophils from healthy subjects but not in neutrophils from severe asthma and COPD, showing insensitivity to the anti-inflammatory effects of corticosteroid. Similar results have been observed by our group in neutrophils from COPD patients^25^. In this work, neutrophils were isolated from severe asthma and COPD patients who showed high levels of peripheral neutrophils and less than 100 eosinophils/µl, and presented an uncontrolled severe asthma and COPD besides an optimal treatment. Unlike fluticasone propionate, the pan-JAK inhibitor LAS194046 showed similar efficacy inhibiting IL-8 and MMP9 release in neutrophils form healthy subjects and COPD patients and in a lesser extent in severe asthma. Similar effects were observed in neutrophils from COPD sputum, showing higher potency and efficacy of LAS194046 inhibiting IL-8 and MMP9 release, than fluticasone propionate. Sputum neutrophils from severe asthma patients were not tested in this work due to the difficulty of obtaining this type of sample, thus representing a limitation of this study.

To our knowledge, this is the first report that studied the expression of different JAK isoforms in neutrophils from healthy, severe asthma and COPD patients. The elevated levels of JAK2 and JAK3 in neutrophils from COPD and severe asthma patients could explain the efficacy of LAS194046 inhibiting IL-8 and MMP9 release, as well as the inhibition of ROS production that we observed. In this regard, it has been shown that JAK/STAT inhibition reduces the expression of NOX1 and NOX4 in vascular smooth muscle cells, thus reducing NOX-dependent oxidative stress^31^, and that tofacitinib inhibits NOX5-dependent ROS production in monocyte-dendritic cells^32^. In this work, LAS194046 reduced superoxide anion production in neutrophils from severe asthma and COPD patients which is in line with previous results in other cell types. In addition, it is known that LPS promotes the production of ROS in neutrophils^33^, and that ROS can phosphorylate JAK2/STAT3^34^ as well as different MAPK, P38 and JNK in macrophages^35^, supporting the anti-inflammatory efficacy of pan-JAK inhibitors in a context of elevated oxidative stress.

Although natural ligands belonging to T2 inflammation, such as IFNγ, IL-5, IL-3 and IL-13 activates JAK/STAT signaling in innate immunity and can be inhibited using pan-JAK inhibitors^14^, other common bacterial mediators present in COPD and severe asthma, such as LPS, can trigger JAK2 and STAT3 phosphorylation in macrophages^35,36^. In addition it has been described that JAK2 phosphorylation enhances IL-8 secretion^34,37^ that could contribute to enhance neutrophilic inflammation, since IL-8 is one of the most potent neutrophil chemoattractant^38,39^. In the same way, phosphorylated JAK2 can also phosphorylate ERK1/2 thus increasing the inflammatory response^40^. In this work we confirmed that LPS phosphorylates JAK2/STAT3, ERK1/2 and P38 in neutrophils from severe asthma and COPD patients which were attenuated by LAS194046, showing synergic inhibitory effects when combined with fluticasone propionate. Therefore, the use of pan-JAK inhibitors in severe asthma and COPD could inhibit both, T2 and non T2 inflammation, which may be of potential value to counteract the impaired anti-inflammatory effects of corticosteroids in severe asthma and COPD. In fact, low concentrations of LAS194046 combined with sub-effective concentrations of fluticasone propionate showed additive/synergic effects reducing IL-8, MMP9 and superoxide anion secretion in neutrophils from severe asthma and COPD patients, thus improving the corticosteroid insensitivity.

Corticosteroids mediates part of their anti-inflammatory effects through the activation of GRE gene regions, thus increasing the expression of corticosteroid dependent anti-inflammatory genes. In this work, transfected GRE reporter assay in bronchial epithelial cells showed synergic effects activating GRE signal when fixed concentration of fluticasone propionate 1 nM was combined with growing concentrations of LAS194046 suggesting a potentiation of the corticosteroid anti-inflammatory properties.

To explain the impaired anti-inflammatory effects of corticosteroids we analyzed the basal expression of mediators of corticosteroid efficacy such as GRα, MKP1 and CD200, as well as the expression of corticosteroid negative regulators such as GRβ and PI3Kδ^23,26^. MKP1 is a mitogen-activated protein kinase phosphatase whose expression is increased by corticosteroids and induces the degradation/de-phosphorylation of several kinases such as ERK1/2 and p38, thus increasing the anti-inflammatory properties of corticosteroids^41^. CD200 is a glucocorticoid-inducible gene that blunts macrophage activation and reduces acute exacerbations of COPD. Pharmacological upregulation of CD200 on airway cells in COPD could attenuate inflammation and reduce exacerbation frequency^42^. In this work, both MKP1 and CD200 expression were significantly lower in neutrophils of severe asthma and COPD patients than in neutrophils from healthy subjects. Furthermore, low concentration of LAS194046 increased MKP1, which may explain the additive/synergic effects of the pan-JAK inhibitor and corticosteroid combination on the suppression of p-ERK1/2 and p-P38 protein expression observed in this work.

The oxidative stress observed in severe asthma and COPD can increase the activation of PI3Kδ which downregulates the histone deacetylase 2 (HDAC2)^43^. Mechanistically, HDAC2 is considered to translate some of the anti-inflammatory effects of corticosteroids by regulating the transcription and protein expression of some of the critical anti-inflammatory mediators downstream of corticosteroid receptor signaling^44^. Previous findings provided by our group indicate that PI3Kδ is elevated and HDAC2 downregulated in neutrophils from COPD patients^25^, combined with elevated levels of non-functional GRβ. In this work we extended these observations to severe asthma patients, thus providing a more complete explanation of the impaired effects of corticosteroids in severe asthma. In this regard, the combination of LAS194046 and fluticasone propionate showed synergic effects reducing PI3Kδ expression and activity. However, this in vitro study has an evident limitation. Since both COPD and asthma are heterogeneous in disease pathogenesis, the small number of subjects included in the in vitro experimental procedures might be not sufficiently representative to extract solid conclusions to all severe asthma and COPD patients, as can be observed in differences in superoxide anion production of different donors between Figs. 3 and 5 of this manuscript.

In summary, the in vitro data provided in this study shows that the pan-JAK inhibitor LAS194046 reduces neutrophil non-T2 inflammatory response and potentiates the impaired anti-inflammatory effects of corticosteroids in neutrophils form severe asthma and COPD patients.

## Supplementary Information

Supplementary Information.

## Data Availability

The datasets used and/or analyzed during the current study are available from the corresponding author on reasonable request.

## References

[1] Mei D, Tan WSD, Wong WSF (2019). Pharmacological strategies to regain steroid sensitivity in severe asthma and COPD. Curr. Opin. Pharmacol..

[2] Menzies-Gow A, Szefler SJ, Busse WW (2021). How do asthma biologics relate to remission for asthma?. J. Allergy Clin. Immunol. In Pract..

[3] Eger K, Kroes JA, Ten Brinke A, Bel EH (2021). Long-term therapy response to anti-IL-5 biologics in severe asthma-a real-life evaluation. J. Allergy Clin. Immunol. In Pract..

[4] Kroes JA, Zielhuis SW, van Roon EN, Ten Brinke A (2020). Prediction of response to biological treatment with monoclonal antibodies in severe asthma. Biochem. Pharmacol..

[5] Wenzel SE (2012). Asthma phenotypes: The evolution from clinical to molecular approaches. Nat. Med..

[6] Moore WC (2010). Identification of asthma phenotypes using cluster analysis in the Severe Asthma Research Program. Am. J. Respir. Crit. Care Med..

[7] Brightling C, Greening N (2019). Airway inflammation in COPD: Progress to precision medicine. Eur. Respir. J..

[8] Rane SG, Reddy EP (2000). Janus kinases: Components of multiple signaling pathways. Oncogene.

[9] Murray PJ (2007). The JAK-STAT signaling pathway: Input and output integration. J. Immunol..

[10] Suzuki K (2000). Janus kinase 3 (Jak3) is essential for common cytokine receptor gamma chain (gamma(c))-dependent signaling: Comparative analysis of gamma(c), Jak3, and gamma(c) and Jak3 double-deficient mice. Int. Immunol..

[11] Southworth T (2016). Anti-inflammatory potential of PI3Kdelta and JAK inhibitors in asthma patients. Respir. Res..

[12] Ashino S (2014). Janus kinase 1/3 signaling pathways are key initiators of TH2 differentiation and lung allergic responses. J. Allergy Clin. Immunol..

[13] Calama E (2017). Tofacitinib ameliorates inflammation in a rat model of airway neutrophilia induced by inhaled LPS. Pulm. Pharmacol. Ther..

[14] Calbet M (2019). Novel inhaled Pan-JAK inhibitor, LAS194046, reduces allergen-induced airway inflammation, late asthmatic response, and pSTAT activation in brown Norway rats. J. Pharmacol. Exp. Ther..

[15] Kudlacz E, Conklyn M, Andresen C, Whitney-Pickett C, Changelian P (2008). The JAK-3 inhibitor CP-690550 is a potent anti-inflammatory agent in a murine model of pulmonary eosinophilia. Eur. J. Pharmacol..

[16] Milara J, Juan G, Peiro T, Serrano A, Cortijo J (2012). Neutrophil activation in severe, early-onset COPD patients versus healthy non-smoker subjects in vitro: Effects of antioxidant therapy. Respir. Int. Rev. Thorac. Dis..

[17] Pizzichini E (1996). Indices of airway inflammation in induced sputum: Reproducibility and validity of cell and fluid-phase measurements. Am. J. Respir. Crit. Care Med..

[18] Su Y, Han W, Giraldo C, De Li Y, Block ER (1998). Effect of cigarette smoke extract on nitric oxide synthase in pulmonary artery endothelial cells. Am. J. Respir. Cell Mol. Biol..

[19] Plumb J (2012). Reduced glucocorticoid receptor expression and function in airway neutrophils. Int. Immunopharmacol..

[20] Dalli E (2008). Hawthorn extract inhibits human isolated neutrophil functions. Pharmacol. Res..

[21] Milara J (2013). Bafetinib inhibits functional responses of human eosinophils in vitro. Eur. J. Pharmacol..

[22] Milara J (2016). Non-neuronal cholinergic system contributes to corticosteroid resistance in chronic obstructive pulmonary disease patients. Respir. Res..

[23] Milara J (2018). MUC1 deficiency mediates corticosteroid resistance in chronic obstructive pulmonary disease. Respir. Res..

[24] Kim SH (2019). Evaluation of neutrophil activation status according to the phenotypes of adult asthma. Allergy Asthma Immunol. Res..

[25] Milara J (2014). Roflumilast N-oxide reverses corticosteroid resistance in neutrophils from patients with chronic obstructive pulmonary disease. J. Allergy Clin. Immunol..

[26] Milara J (2019). Mucin 1 deficiency mediates corticosteroid insensitivity in asthma. Allergy.

[27] Shaw DE (2007). Association between neutrophilic airway inflammation and airflow limitation in adults with asthma. Chest.

[28] Uddin M (2010). Prosurvival activity for airway neutrophils in severe asthma. Thorax.

[29] Wenzel SE (1997). Bronchoscopic evaluation of severe asthma. Persistent inflammation associated with high dose glucocorticoids. Am. J. Respir. Crit. Care Med..

[30] Shukla SD (2021). Add-on azithromycin reduces sputum cytokines in non-eosinophilic asthma: An AMAZES substudy. Thorax.

[31] Manea A, Tanase LI, Raicu M, Simionescu M (2010). Jak/STAT signaling pathway regulates nox1 and nox4-based NADPH oxidase in human aortic smooth muscle cells. Arterioscler. Thromb. Vasc. Biol..

[32] Marzaioli V (2020). Monocyte-derived dendritic cell differentiation in inflammatory arthritis is regulated by the JAK/STAT axis via NADPH oxidase regulation. Front. Immunol..

[33] Evani SJ, Karna SLR, Seshu J, Leung KP (2020). Pirfenidone regulates LPS mediated activation of neutrophils. Sci. Rep..

[34] Xu Z, Wu H, Zhang H, Bai J, Zhang Z (2020). Interleukins 6/8 and cyclooxygenase-2 release and expressions are regulated by oxidative stress-JAK2/STAT3 signaling pathway in human bronchial epithelial cells exposed to particulate matter </=2.5 mum. J. Appl. Toxicol..

[35] Kim SH (2017). Chlorogenic acid suppresses lipopolysaccharideinduced nitric oxide and interleukin1beta expression by inhibiting JAK2/STAT3 activation in RAW2647 cells. Mol. Med. Rep..

[36] Lee JJ (2013). Toll-like receptor 4-linked Janus kinase 2 signaling contributes to internalization of *Brucella**abortus* by macrophages. Infect. Immun..

[37] Abraham RT (2012). Chemokine to the rescue: Interleukin-8 mediates resistance to PI3K-pathway-targeted therapy in breast cancer. Cancer Cell.

[38] Milara J (2009). Sphingosine-1-phosphate increases human alveolar epithelial IL-8 secretion, proliferation and neutrophil chemotaxis. Eur. J. Pharmacol..

[39] Schalper KA (2020). Elevated serum interleukin-8 is associated with enhanced intratumor neutrophils and reduced clinical benefit of immune-checkpoint inhibitors. Nat. Med..

[40] Barrios-Correa AA, Estrada JA, Contreras I (2018). Leptin signaling in the control of metabolism and appetite: Lessons from animal models. J. Mol. Neurosci..

[41] Ralph JA (2010). Identification of NURR1 as a mediator of MIF signaling during chronic arthritis: Effects on glucocorticoid-induced MKP1. Am. J. Pathol..

[42] Snelgrove RJ (2008). A critical function for CD200 in lung immune homeostasis and the severity of influenza infection. Nat. Immunol..

[43] Marwick JA (2009). Inhibition of PI3Kdelta restores glucocorticoid function in smoking-induced airway inflammation in mice. Am. J. Respir. Crit. Care Med..

[44] Barnes PJ (2010). Mechanisms and resistance in glucocorticoid control of inflammation. J. Steroid Biochem. Mol. Biol..

