# Supplementary material for: The pan-JAK inhibitor LAS194046 reduces neutrophil activation from severe asthma and COPD patients in vitro
Source: Sci Rep. 2022 Mar 24;12:5132. doi: 10.1038/s41598-022-09241-6 (PMC8948298; doi:10.1038/s41598-022-09241-6)
Supplement: Supplementary file 1 — Supplementary Information. [file 41598_2022_9241_MOESM1_ESM.pdf]

SUPPLEMENTARY INFORMATION

TITLE

The pan-JAK Inhibitor LAS194046 reduces neutrophil activation from severe asthma and COPD patients *in vitro*

Authors
Javier Milara[1,2,3]* Beatriz Ballester[1,3]*, Alfredo de Diego[4], Marta Calbet,[5] Isabel Ramis,[5] Montserrat Miralpeix,[5] Julio Cortijo[1,3,6]

Affiliations
[1]Department of Pharmacology, Faculty of Medicine, University of Valencia, Spain
[2]Pharmacy Unit, University General Hospital Consortium, Valencia, Spain
[3]CIBERES, Health Institute Carlos III, Valencia, Spain
[4]Respiratory Unit, University and Polytechnic La Fe Hospital, Valencia, Spain
[5]Almirall, R&D Centre, Barcelona, Spain
[6]Research and teaching Unit, University General Hospital Consortium, Valencia, Spain
*Both authors contributed equally to this work

Supplementary tables

Table S1: Clinical features of COPD patients. COPD: chronic obstructive pulmonary disease; FEV1: forced expiratory volume in one second; FVC: forced vital capacity; Pack-yr = 1 year smoking 20 cigarettes-day. Data are mean ± SD. * *p*< 0.05 related to Healthy subjects

	Healthy (n=23)	COPD (n= 23)
Age, yr	66 ± 6	65.5 ±11
Sex (M/F)	13/10	18/5
Tobacco consumption, pack-yr	0	36.2±11
FEV1, % pred	99 ± 6	32.1 ± 9*
FVC, % pred	96±3	90.1±11
FEV1/FVC %	96±7	51.2±4*
Receiving inhaled steroids, no.	0	19*
Receiving theophylline, no.	0	0
Receiving long-acting b2-agonist, no.	0	19*
Receiving anticholinergics, no.	0	18*
Receiving roflumilast, no.	0	4*
Total peripheral blood neutrophils	4.2 ± 0.3 cells x 10^9/L	8.2 ± 1.3 cells x 10^9/L*
Total peripheral blood eosinophils	0.09 ± 0.01 cells x 10^9/L	0.08± 0.02 cells x 10^9/L

Table S2: Clinical features of asthma patients. FEV1: forced expiratory volume in one second; FVC: forced vital capacity; Pack-yr = 1 year smoking 20 cigarettes-day. Data are mean ± SD. * p< 0.05 related to Healthy subjects. na: not apply

	Healthy (n=23)	Asthma (n= 21)
Age, yr	66 ± 6	55 ±10
Sex (M/F)	13/10	13/8
Tobacco consumption (yes/no)	0	5/21
Tobacco consumption, pack-yr	0	7.6±15
FEV1, before bronchodilation (%pred)	na	64 ± 27.3
FEV1, after bronchodilation (%pred)	na	74 ± 24.6
FEV1/FVC %	na	57.4 ± 18.9
Asthma duration (years)	na	23.2 ± 15.2
Receiving inhaled steroids, no.	0	21
Fluticasone (mg)<br>*Budesonide (mg)*		750 ± 273.9<br>512 ± 165.2
Receiving oral steroids, (yes/no).	0	6/14
oral steroids dose/day (mg)		17.5 ± 10.8
Atopy (yes/no)	0	12/9
Reversibility (yes/no)	na	12/9
Reversibility (ml)		367.9 ± 145.5
Total peripheral blood neutrophils	4.2 ± 0.3 x 10^9 cells /L	6.4 ± 1.2 x 10^9 cells /L*
Total peripheral blood eosinophils	0.09 ± 0.01 x 10^9 cells /L	0.085± 0.03 x 10^9 cells /L*

Table S3. Maximal percentage of inhibition of IL-8 and MMP-9 release from isolated peripheral blood neutrophils from healthy subjects, severe asthma and COPD patients. Neutrophils were incubated with LAS194046 (0.01nM-1µM) or fluticasone propionate (FLUT; 0.1nM-1µM) in response to LPS (1µg/ml). Sputum neutrophils from COPD patients were stimulated with cigarette smoke extract (CSE 5%). The levels of IL-8 and MMP-9 in the cell supernatant were determined, and the maximal % of inhibition of IL-8 and MMP-9 release by LAS194046 and FLUT were calculated. Values are mean ± SD of 3 independent experiments run in triplicate. *p < 0.05 *vs* healthy Subjects values.

Neutrophils	Stimulus	Treatment	Maximal % inhibition		
			Healthy	COPD	Asthma
IL-8 (blood)	LPS	LAS194046	70.54±15	58.95±22.64	45.1±7.5*
		FLUT	99.01±2.26	66.57±7.45*	50.77±5.43*
MMP-9 (blood)	LPS	LAS194046	98.1±14.86	97.68±3.29	63.44±2.44*
		FLUT	90.85±7.66	62.09±3.79*	42.72±6.25*
IL-8 (sputum)	CSE	LAS194046		57.27±10.13*	
		FLUT		40.12±10.30	
MMP-9 (sputum)	CSE	LAS194046		74.34±3.09*	
		FLUT		42.14±9.23	

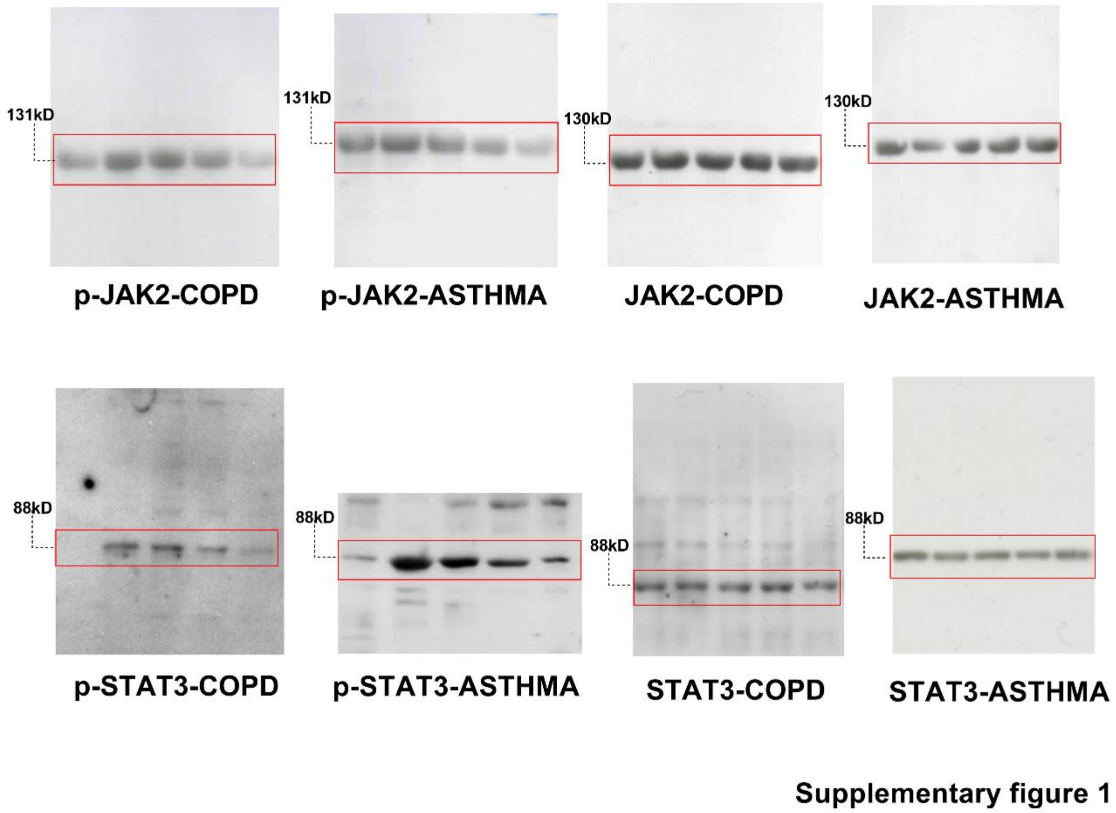

Supplementary figure S1. Uncropped Western blots from Figure 9. Immunoblots as shown in Fig. 9A. Cropped inserts shown in main figures are demarcated by red box. Samples were derived from the same experiment and blots were processed in parallel.

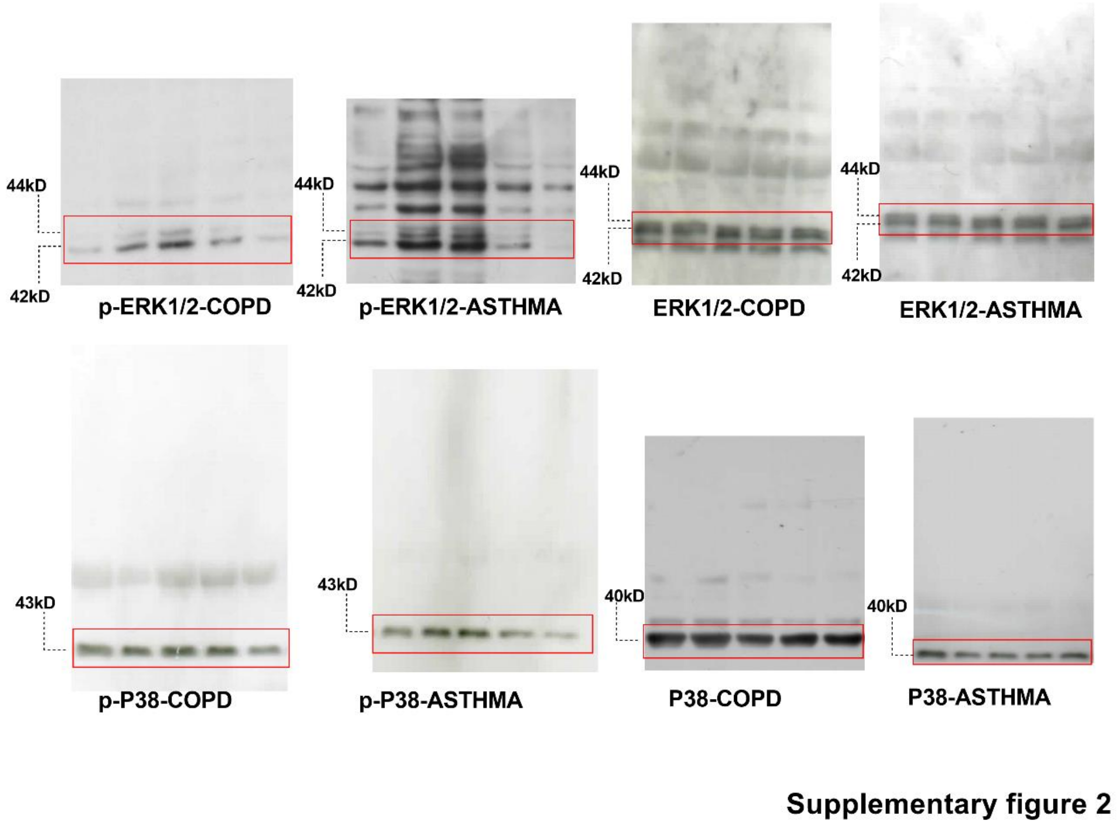

Supplementary figure S2. Uncropped Western blots from Figure 9. Immunoblots as shown in Fig. 9B. Cropped inserts shown in main figures are demarcated by red box. Samples were derived from the same experiment and blots were processed in parallel.
